# Differences in postoperative knee function based on concomitant treatment of lateral meniscal injury in the setting of primary ACL reconstruction

**DOI:** 10.1186/s12891-023-06867-z

**Published:** 2023-09-15

**Authors:** Janina Kaarre, Zachary J. Herman, Fabian Persson, Jonas Olsson Wållgren, Eduard Alentorn-Geli, Eric Hamrin Senorski, Volker Musahl, Kristian Samuelsson

**Affiliations:** 1 Department of Orthopaedics, Institute of Clinical Sciences, Sahlgrenska Academy University of Gothenburg, Göteborgsvägen 31, 43180 Gothenburg, Mölndal, Sweden; 2 Sahlgrenska Sports Medicine Center, Gothenburg, Sweden; 3 Department of Orthopaedic Surgery, UPMC Freddie Fu Sports Medicine Center, University of Pittsburgh Medical Center, Pittsburgh, PA USA; 4 Department of Orthopaedics, the NU Hospital Group, Trollhättan, Sweden; 5 Instituto Cugat, Hospital Quironsalud Barcelona, Barcelona, Spain; 6 Mutualidad de Futbolistas Españoles - Delegación Catalana, Barcelona, Spain; 7 Fundación García Cugat, Barcelona, Spain; 8 Unit of Physiotherapy, Department of Health and Rehabilitation, Institute of Neuroscience and Physiology, Sahlgrenska Academy, University of Gothenburg, Gothenburg, Sweden; 9 Department of Orthopaedics, Sahlgrenska University Hospital, Mölndal, Sweden

**Keywords:** Lateral meniscal repair, Lateral meniscal resection, KOOS

## Abstract

**Background:**

Concomitant lateral meniscal (LM) injuries are common in acute anterior cruciate ligament (ACL) ruptures. However, the effect of addressing these injuries with various treatment methods during primary ACL reconstruction (ACLR) on patient-reported outcomes (PROs) is unknown. Therefore, the purpose of this study was to compare postoperative Knee injury and Osteoarthritis Outcome Score (KOOS) at 2-, 5-, and 10-years after isolated primary ACLR to primary ACLR with various treatment methods to address concomitant LM injury.

**Methods:**

This study was based on data from the Swedish National Knee Ligament Registry. Patients ≥ 15 years with data on postoperative KOOS who underwent primary ACLR between the years 2005 and 2018 were included in this study. The study population was divided into five groups: 1) Isolated ACLR, 2) ACLR + LM repair, 3) ACLR + LM resection, 4) ACLR + LM injury left in situ, and 5) ACLR + LM repair + LM resection. Patients with concomitant medial meniscal or other surgically treated ligament injuries were excluded.

**Results:**

Of 31,819 included patients, 24% had LM injury. After post hoc comparisons, significantly lower scores were found for the KOOS Symptoms subscale in ACLR + LM repair group compared to isolated ACLR (76.0 vs 78.3, *p* = 0.0097) and ACLR + LM injury left in situ groups (76.0 vs 78.3, *p* = 0.041) at 2-year follow-up. However, at 10-year follow-up, no differences were found between ACLR + LM repair and isolated ACLR, but ACLR + LM resection resulted in significantly lower KOOS Symptoms scores compared to isolated ACLR (80.4 vs 82.3, *p* = 0.041).

**Conclusion:**

The results of this study suggest that LM injury during ACLR is associated with lower KOOS scores, particularly in the Symptoms subscale, at short- and long-term follow-up. However, this finding falls below minimal clinical important difference and therefore may not be clinically relevant.

**Level of Evidence:**

III.

**Supplementary Information:**

The online version contains supplementary material available at 10.1186/s12891-023-06867-z.

## Introduction

Concomitant, traumatic lateral meniscal (LM) tears are frequently found in patients with acute anterior cruciate ligament (ACL) injuries [1, 2], and have been associated with increased knee instability, risk of cartilaginous defects, and later development of osteoarthritis [1, 3–7]. Meniscal tears in the setting of ACL reconstruction (ACLR) can be treated operatively with repair or partial meniscectomy or nonoperatively depending on patients’ intended future knee function and the type of the tear [8]. Studies have reported decreased long term risk of osteoarthritis development as well as increased activity levels and quality of life scores when patients are treated with meniscal repair instead of partial meniscal resection [1, 9–14]. However, as meniscal repair has been associated with more extensive postoperative rehabilitation [15] and higher revision risk than partial meniscectomy [13, 16], partial meniscal resection or non-operative treatment may be necessary in irreparable meniscal injuries or in patients wishing for faster return to sport [3, 17].

Research has variable results when comparing short-term subjective knee outcomes in patients undergoing medial or LM repair versus partial meniscal resection in the setting of ACLR [18–20]. At 6 months to 1 year postoperatively, patients undergoing concomitant meniscal resection with ACLR have reported comparable subjective knee function to isolated ACLR [18]. Yet, patients undergoing meniscal repair with ACLR reported slightly worse subjective knee function in the same postoperative time periods [18]. However, at 2-year follow-up, studies report that clinical outcomes of concomitant meniscal repair with ACLR are more comparable to isolated ACLR than are outcomes of ACLR with meniscal resection [19, 20]. It is unclear what effect the treatment of meniscal pathology, particularly LM injury, in the setting of ACLR has on mid- to long-term patient-reported outcomes (PROs) of subjective knee function. Previous studies have reported that concomitant LM injury in the setting of ACLR places a patient at higher risk of persistent knee laxity and thus, higher risk of subsequent injuries [21, 22]. However, subjective knee function outcomes following ACLR with concomitant LM treatment are less studied. Thus, an increased understanding of the outcomes of several treatment methods of LM injury in the setting of primary ACLR would improve preoperative planning, postoperative outcomes, and patient education. As such, the aim of this study was to compare the effect of primary isolated ACLR versus primary ACLR with concomitant LM injury (addressed with repair, resection, a combination of repair and resection, or left in situ) on the Knee injury and Osteoarthritis Outcome Score (KOOS) at 2-, 5-, and 10-years postoperatively.

## Methods

This registry cohort study was performed in accordance with the Declaration of Helsinki, and approved by the Regional Ethical Board in Stockholm, Sweden (2011/337–31/3), and the Swedish Ethical Review Authority (2022–00913-01). Strengthening the Reporting of Observational Studies in Epidemiology guidelines were used to present this study [23].

This observational cohort study included data obtained from the SNKLR, in which 90% of all ACLR performed annually in Sweden are reported. The SNKLR is a national database that was established in January 2005 and uses a web-based protocol consisting of two parts: a surgeon reported section and a patient reported section [24]. While demographical (sex, age, date of injury, activity at time of injury) and surgical data (surgery type, graft type, concomitant injuries, previous surgery) are reported by the surgeon, patients are asked to fill out the web-based questionnaires regarding to knee function (KOOS, EQ-5D) preoperatively and at 1-, 2-,5 and 10-year follow-ups. Revision surgeries and reoperations for other reasons are registered separately and subsequently correlated with primary ACL surgeries. Furthermore, the participation in the registry is optional and patients are given option to request exclusion if research participation would not be desired. More detailed description of the registry can be found in previous literature [25].

### Study population and data collection

Patients aged ≥ 15 years who underwent primary ACLR between 2005 and 2018 and completed postoperative KOOS questionnaires were included in this study. Exclusion criteria were any prior knee surgery, concomitant fracture, concomitant medial meniscal injury, concomitant posterior cruciate ligament injury, or neurovascular injury. Also, patients undergoing double bundle ACLR and patients receiving surgical treatment for concomitant collateral ligament injury were excluded.

The study population was divided into five different groups to determine the effect of different treatments of LM injury in the setting of ACL reconstruction: 1) Isolated ACLR (patients undergoing isolated ACLR without concomitant LM injury or procedure), 2) ACLR + LM repair (patients undergoing ACLR with concomitant LM repair), 3) ACLR + LM resection (patients undergoing ACLR with concomitant LM resection), 4) ACLR + LM injury left in situ (patients undergoing ACLR with nonoperatively treated concomitant LM injury), and 5) ACLR + LM repair + LM resection (patients undergoing ACLR with concomitant LM repair + LM resection).

Patient data (sex, age, body mass index [BMI]), injury (activity at time of injury, concomitant injury) and surgical characteristics (graft type, time from injury to surgery) were extracted from the registry. The activity at the time of injury was divided into six groups: 1) pivoting sport (American football, rugby, basketball, dancing, floorball, gymnastics, handball, ice hockey/bandy, martial arts, racket sports, soccer, volleyball, wrestling); 2) non-pivoting sport (cross-country skiing, cycling, horseback riding, motocross/endure, skateboarding, snowboarding, and surfing/wakeboarding); 3) alpine/skiing; 4) other physical activity (other recreational sport, exercise, trampoline); 5) traffic related; and 6) other (other outdoor activity and work).

### Outcome measures

The main outcome of interest for this study was the patient-reported postoperative KOOS at 2-, 5-, and 10-years after the primary ACLR. The KOOS consists of 5 subscales (Pain, Other Symptoms, Function in Sport and Recreation [Sport/Rec], Knee-Related Quality of Life [QoL], and Activities of Daily Living [ADL], including questions related to knee function. Patients are asked whether they have any pain, other symptoms including restricted range of motion or mechanical symptoms, difficulties with performing physical activities and activities of daily living or whether they have needed to make any life changes due to their current knee status. All the questions are ranged from 0 to 4 on 5-point Likert Scale, and each subscale is scaled from 0 to 100, where the latter indicates the best outcome [26]. Even though the KOOS was originally created to assess patients with knee osteoarthritis, it has also been later used in other orthopaedic knee conditions, including ACLR, to assess outcomes following a specific treatment approach [26, 27].

### Statistical analyses

The SAS System for Windows software (version 9.4, SAS Institute, North Carolina, USA) was used to perform the statistical analyses. Count (n) and proportion (%) were used to present categorical variables, while mean with standard deviation (SD) and median with minimum and maximum were used to present continuous and ordinal variable, respectively. For comparisons between groups, including post-hoc analysis, the Kruskal–Wallis test was used for continuous variables, while for pairwise comparisons between groups either the Fisher’s non-parametric permutation test or the Mann–Whitney U-test was used for continuous variables due to non-parametric data. Logistic regression analysis was used for adjusting for the concomitant cartilaginous injuries, considering their previously reported influence on postoperative outcomes [28]. All significance tests were conducted at the 5% significance level.

## Results

### Baseline characteristics

**Fig. 1:**
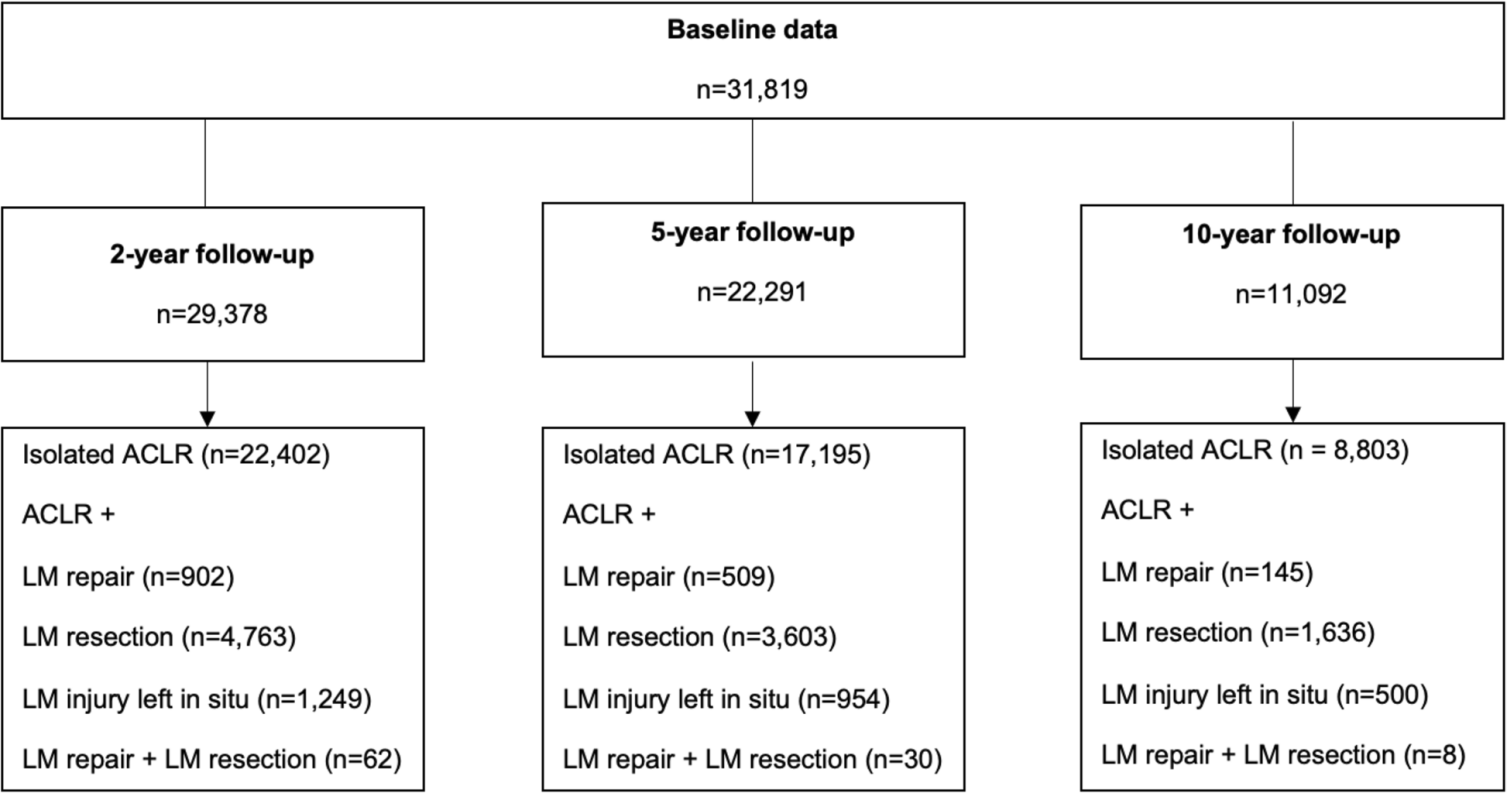
Flow Chart on Patient Enrollment ACLR = anterior cruciate ligament reconstruction; LM = lateral meniscus

Table 1 presents baseline characteristics. The majority of patients in the study underwent isolated ACLR (24,144). Of the patients with concomitant LM injury, more patients underwent ACLR + LM resection (*n* = 5,152) compared to ACLR + LM repair (*n* = 1,099) or ACLR + LM injury left in situ (*n* = 1,342) (Fig. 1). Lateral meniscal injuries were treated with combined treatment (ACLR + LM repair + LM resection) in the smallest population of patients (*n* = 82).

**Table 1:** Baseline characteristics of the included patients

**Variable**	**Total** **(*n* = 31,819)**	**Isolated ACLR** **(*n* = 24,144)**	**ACLR + LM repair** **(*n* = 1,099)**	**ACLR + LM resection (*n* = 5,152)**	**ACLR + LM injury left in situ** **(*n* = 1,342)**	**ACLR + LM repair + LM resection** **(*n* = 82)**
**Age at the time of injury (years)**	25.6 ± 9.6 23 (1–70)	26.0 ± 9.8 23 (1–70)	22.6 ± 8.3 20 (1–57)	24.7. ± 8.7 22 (7–64)	24.0 ± 8.5 21 (11–58)	21.3 ± 7.2 19 (11–50)
**Age at the time of surgery (year)**	27.1 ± 9.9 25 (15–71)	27.6 ± 10.1 25 (15–71)	23.9 ± 8.7 21 (15–58)	26.2 ± 9.1 24 (15–66)	25.3 ± 8.8 23 (15–60)	22.4 ± 7.4 19.5 (15–50)
**Sex (male)**	17,854 (56.1)	12,925 (53.5)	622 (56.6)	3,491 (67.8)	756 (56.3)	60 (73.2)
**BMI (kg/m**^**2**^**)**	24.5 ± 3.3 24.2 (15.4–49.8)	24.5 ± 3.3 24.1 (15.4–49.8)	23.8 ± 2.8 23.4 (17.4–35.4)	24.8 ± 3.2 24.4 (16–42.7)	24.5 ± 3.3 23.9 (17.8–44.1)	24.0 ± 2.9 23.6 (19–29.6)
**Smoking (yes)**	789 (2.5)	608 (2.5)	26 (2.4)	118 (2.3)	34 (2.5)	3 (3.7)
**Time from injury to surgery (months)**	17.0 ± 30.8 7.5 (0–458.4)	17.4 ± 31.4 7.7 (0–458.4)	13.6 ± 27.0 5.9 (0.1–247.9)	16.9 ± 29.7 7.2 (0.1–434.9)	14.6 ± 27.2 7.2 (0.1–358.9)	10.4 ± 19.6 5.3 (0.8–140.8)
**ACL graft (yes)**						
Patellar tendon autograft	1,848 (5.8)	1,407 (5.8)	60 (5.5)	310 (6.0)	67 (5.0)	4 (4.9)
Semitendinosus autograft	28,977 (91.1)	22,022 (91.2)	966 (88.9)	4,688 (91.0)	1,238 (92.3)	63 (76.8)
Quadriceps tendon autograft	525 (1.6)	355 (1.5)	49 (4.5)	89 (1.7)	18 (1.3)	14 (17.1)
Allograft	62 (0.2)	40 (0.2)	8 (0.7)	11 (0.2)	3 (0.2)	0 (0.0)
Direct suture/synthetic/other	52 (0.2)	38 (0.2)	4 (0.4)	4 (0.1)	5 (0.4)	1 (1.2)
**Concomitant injury except meniscal injury (yes)**	7,962 (25.0)	5,524 (22.9)	334 (30.4)	1,669 (32.4)	391 (29.1)	44 (53.7)
**Cartilaginous injury (yes)**						
Lateral femoral condyle	1,580 (5.0)	842 (3.5)	87 (7.9)	542 (10.5)	95 (7.1)	14 (17.1)
Medial femoral condyle	4,241 (13.3)	3,096 (12.8)	167 (15.2)	789 (15.3)	166 (12.4)	23 (28.0)
Lateral patella	702 (2.2)	494 (2.0)	32 (2.9)	145 (2.8)	28 (2.1)	3 (3.7)
Medial patella	1,274 (4.0)	939 (3.9)	37 (3.4)	223 (4.3)	68 (5.1)	7 (8.5)
Lateral tibial plateau	1,768 (5.6)	1,031 (4.3)	93 (8.5)	515 (10.0)	114 (8.5)	15 (18.3)
Medial tibial plateau	1,066 (3.4)	815 (3.4)	27 (2.5)	193 (3.7)	28 (2.1)	3 (3.7)
Trochlea	752 (2.4)	523 (2.2)	38 (3.5)	157 (3.0)	30 (2.2)	4 (4.9)
**Collateral ligament injury (yes)**						
LCL	252 (0.8)	161 (0.7)	16 (1.5)	55 (1.1)	19 (1.4)	1 (1.2)
MCL	1,208 (3.8)	821 (3.4)	73 (6.6)	214 (4.2)	92 (6.9)	8 (9.8)
**PLC injury (yes)**	24 (0.1)	16 (0.1)	2 (0.2)	6 (0.1)	0 (0.0)	0 (0.0)
**Activity at the time of injury (yes)**						
Alpine/skiing	4,742 (14.9)	3,846 (15.9)	158 (14.4)	543 (10.5)	185 (13.8)	10 (12.2)
Pivoting sport	21,491 (67.6)	15,926 (66.0)	786 (71.5)	3,759 (73.0)	956 (71.2)	64 (78.0)
Non-pivoting sport	1,305 (4.1)	958 (4.0)	45 (4.1)	235 (4.6)	65 (4.8)	2 (2.4)
Other physical activity	1,168 (3.7)	918 (3.8)	29 (2.6)	173 (3.4)	46 (3.4)	2 (2.4)
Traffic-related	494 (1.6)	368 (1.5)	20 (1.8)	85 (1.6)	19 (1.4)	2 (2.4)
Other	2,558 (8.0)	2,072 (8.6)	60 (5.5)	354 (6.9)	70 (5.2)	2 (2.4)

Values are given as n (%) and mean ± SD or median (minimum–maximum) for categorical and continuous as well as ordinal variables, respectively. The sums may vary to because of missing values. The variables with missing values, n (%) of the total sample were Age at the time of injury 714 (2.2), BMI 15,262 (50.0), Smoking 14,997 (47.1), Time from injury to surgery 753 (2.4), Cartilaginous injury 24,901 (78.2), ACL graft 355 (1.1), and Activity at the time of injury 61 (0.2)

Pivoting sport (American football/rugby, basketball, dancing, floorball, gymnastics, handball, ice hockey/bandy, martial arts, racket sports, soccer, volleyball, wrestling); Non-pivoting sport (cross-country skiing, cycling, horseback riding, motocross/endure, skateboarding, snowboarding, and surfing/wakeboarding); Alpine/skiing; Other physical activity (other recreational sport, exercise, trampoline); Traffic related, and Other (other outdoor activity and work)

*ACL* Anterior cruciate ligament, *ACLR* Anterior cruciate ligament reconstruction, *BMI* Body mass index, *LCL* Lateral collateral ligament, *LM* Lateral meniscus, *MCL* Medial collateral ligament, *PLC* Posterior lateral corner, *SD* Standard deviation

### Postoperative KOOS by Lateral Meniscal Status 2 years after ACLR

Table 2 shows KOOS results at 2-year follow-up across LM treatment methods. The overall 2-year follow-up data was available for 29,378 patients (92.3%). No significant differences were found between isolated ACLR or the four LM treatment methods for four of five (4/5) KOOS subscales: Sports/Rec, Pain, ADL, and QoL. Significant difference was seen for the KOOS Symptoms subscale (*p* = 0.0067). When post hoc comparisons were performed across LM status for the KOOS Symptom subscale, ACLR + LM repair (76.0 ± 18.3) resulted in significantly lower KOOS Symptoms scores compared to isolated ACLR (78.3 ± 17.9,* p* = 0.0097) and ACLR + LM injury left in situ (78.3 ± 17.4, *p* = 0.041). All other post-hoc comparisons were statistically insignificant (Table 3).

**Table 2:** Post-operative KOOS at 2-year follow-up by lateral meniscal status

	**Isolated ACLR** **(*n* = 22,402)**	**ACLR + LM repair** **(*n* = 902)**	**ACLR + LM resection** **(*n* = 4,763)**	**ACLR + LM injury left in situ** **(*n* = 1,249)**	**ACLR + LM repair + LM resection** **(*n* = 62)**	***P***
**Sport/Rec**	66.0 ± 27.3 70 (0–100) *n* = 11,109	65.9 ± 25.8 70 (0–100) *n* = 425	65.6 ± 26.9 70 (0–100) *n* = 2,200	66.2 ± 26.6 70 (0–100) *n* = 600	73.3 ± 18.5 70 (40–100) *n* = 21	0.75
**Symptoms**	78.3 ± 17.9 82.1 (0–100) *n* = 11,114	76.0 ± 18.3 78.6 (7.1–100) *n* = 426	77.5 ± 17.9 82.1 (3.6–100) *n* = 2,202	78.3 ± 17.4 82.1 (3.6–100) *n* = 599	75.3 ± 16.5 78.6 (42.9–100) *n* = 21	**0.0067**
**Pain**	84.8 ± 16.0 88.9 (0–100) *n* = 11,113	84.7 ± 15.9 88.9 (8.3–100) *n* = 426	85.1 ± 15.6 88.9 (2.8–100) *n* = 2,201	85.1 ± 15.3 88.9 (11.1–100) *n* = 600	89.0 ± 11.4 91.7 (69.4–100) *n* = 21	0.80
**ADL**	91.3 ± 13.5 97.1 (0–100) *n* = 11,114	92.4 ± 12.6 97.1 (10.3–100) *n* = 425	91.5 ± 13.3 97.1 (0–100) *n* = 2,201	91.8 ± 13.4 97.1 (0–100) *n* = 600	94.9 ± 6.3 95.6 (75–100) *n* = 21	0.67
**QoL**	61.4 ± 24.3 62.5 (0–100) *n* = 11,111	60.5 ± 22.9 62.5 (0–100) *n* = 425	60.3 ± 23.9 62.5 (0–100) *n* = 2,199	60.3 ± 24.5 62.5 (0–100) *n* = 600	62.8 ± 23.8 56.3 (31.3–100) *n* = 21	0.18

Values are mean ± SD and median (minimum–maximum)

The total amount of follow-up data may vary within the KOOS subgroups. Thus, the numbers in the headline refer to the total count of patients corresponding follow-up data. Within the various KOOS subgroups, the numbers encompass the count of patients who completed their respective KOOS subgroup questionnaires

*ACL* Anterior cruciate ligament, *ACLR* Anterior cruciate ligament reconstruction, *ADL* Activities of daily living, *KOOS* Knee injury and Osteoarthritis Outcome Score, *LCL* Lateral collateral ligament, *LM* Lateral meniscus, *MCL* Medial collateral ligament, *PLC* Posterior lateral corner, *QoL* Quality of life, *SD* Standard deviation, *Sport/Rec* Function in Sport and Recreation

**Table 3:** Post hoc comparisons: Postoperative KOOS between groups at 2- year follow-up

KOOS Sport/Rec 2 years				
	**ACLR + LM repair**	**ACLR + LM resection**	**ACLR + LM injury left in situ**	**ACLR + LM repair + LM resection**
**Isolated ACLR**	0.94	0.86	0.75	0.18
**ACLR + LM repair**	-	0.98	0.85	0.19
**ACLR + LM resection**	-	-	0.78	0.18
**ACLR + LM injury left in situ**	-	-	-	0.23
**KOOS Symptoms 2 years**^**a**^				
**Isolated ACLR**	**0.0097**	0.091	1.00	0.48
**ACLR + LM repair**	-	0.10	**0.041**	0.88
**ACLR + LM resection**	-	-	0.36	0.59
**ACLR + LM injury left in situ**	-	-	-	0.44
**KOOS Pain 2 years**				
** Isolated ACLR**	0.96	0.21	0.59	0.20
**ACLR + LM repair**	-	0.63	0.73	0.23
**ACLR + LM resection**	-	-	0.92	0.24
**ACLR + LM injury left in situ**	-	-	-	0.25
**KOOS ADL 2 years**				
**Isolated ACLR**	0.083	0.18	0.29	0.20
**ACLR + LM repair**	-	0.27	0.51	0.37
**ACLR + LM resection**	-	-	0.73	0.24
**ACLR + LM injury left in situ**	-	-	-	0.29
**KOOS QoL 2 years**				
**Isolated ACLR**	0.49	0.095	0.28	0.75
**ACLR + LM repair**	-	0.90	0.87	0.64
**ACLR + LM resection**	-	-	0.95	0.62
**ACLR + LM injury left in situ**	-	-	-	0.68

^**a**^Difference between groups mean (95% CI) and effect size were calculated for the KOOS Symptoms between Isolated ACLR and ACLR + LM repair groups as well as ACLR + LM repair and ACLR LM injury left in situ, respectively. For Isolated ACLR and ACLR + LM repair: 95% CI, 2.35 (0.59; 4.14); Effect size, 0.131. For ACLR + LM repair and ACLR + LM injury left in situ: 95% CI -2.31 (-4.60; -0.08); Effect size, 0.130

*ACLR* Anterior cruciate ligament reconstruction, *ADL* Activities of daily living, *CI* Confidence Interval, *KOOS* Knee injury and Osteoarthritis Outcome Score, *LM* Lateral meniscus, *QoL* Quality of life, *SD* Standard deviation, *Sport/Rec* Function in Sport and Recreation

### Postoperative KOOS by Lateral Meniscal Status 5 years after ACLR

Table 4 shows KOOS results at 5-year follow-up across LM treatment methods. The overall 5-year follow-up data was available for 22,291 patients (70.1%). No significant differences were found between isolated ACLR or the four LM treatment methods for four of five (4/5) KOOS subscales: Sports/Rec, Pain, ADL, and QoL. Significant difference was seen for the KOOS Symptoms subscale (*p* = 0.018). However, when post hoc comparisons were performed across LM status for the KOOS Symptoms subscale, not pairwise comparisons were found to be statistically significant (Table 5).

**Table 4:** Post-operative KOOS at 5-year follow-up by lateral meniscal status

	**Isolated ACLR** **(*n* = 17,195)**	**ACLR + LM repair** **(*n* = 509)**	**ACLR + LM resection** **(*n* = 3,603)**	**ACLR + LM injury left in situ** **(*n* = 954)**	**ACLR + LM repair + LM resection** **(*n* = 30)**	***P***
**Sport/Rec**	67.7 ± 28.0 75 (0–100) *n* = 7,581	67.0 ± 26.9 70 (0–100) *n* = 209	68.1 ± 26.9 75 (0–100) *n* = 1,441	67.3 ± 28.0 75 (0–100) *n* = 387	76.7 ± 26.2 80 (35–100) *n* = 9	0.79
**Symptoms**	80.7 ± 17.9 85.7 (0–100) *n* = 7,588	78.9 ± 18.2 82.1 (28.6–100) *n* = 210	79.8 ± 17.8 85.7 (7.1–100) *n* = 1,441	80.2 ± 18.2 85.7 (3.6–100) *n* = 387	88.5 ± 19.7 96.4 (39.3–100) *n* = 9	**0.018**
**Pain**	86.1 ± 16.0 91.7 (2.8–100) *n* = 7,587	85.9 ± 16.0 91.7 (16.7–100) *n* = 210	86.0 ± 16.1 91.7 (0–100) *n* = 1,440	86.3 ± 15.5 91.7 (11.1–100) *n* = 387	91.7 ± 12.2 97.1 (69.4–100) *n* = 9	0.74
**ADL**	91.9 ± 13.3 97.1 (2.9–100) *n* = 7,588	92.4 ± 12.7 98.5 (42.1–100) *n* = 210	92.0 ± 13.5 98.4 (13.2–100) *n* = 1,441	92.0 ± 12.8 97.1 (27.9–100) *n* = 387	95.8 ± 8.0 100 (80.9–100) *n* = 9	0.70
**QoL**	64.6 (25.0) 68.8 (0–100) *n* = 7,583	64.6 ± 25.0 68.8 (0–100) *n* = 7,583	64.2 ± 24.9 68.8 (0–100) *n* = 1,440	64.0 ± 25.5 68.8 (0–100) *n* = 387	66.7 ± 29.1 75 (25–100) *n* = 9	0.78

Values are mean ± SD and median (minimum–maximum)

The total amount of follow-up data may vary within the KOOS subgroups. Thus, the numbers in the headline refer to the total count of patients corresponding follow-up data. Within the various KOOS subgroups, the numbers encompass the count of patients who completed their respective KOOS subgroup questionnaires

*ACL* Anterior cruciate ligament, *ACLR* Anterior cruciate ligament reconstruction, *ADL* Activities of daily living, *KOOS* Knee injury and Osteoarthritis Outcome Score, *LCL* Lateral collateral ligament, *LM* Lateral meniscus, *MCL* Medial collateral ligament, *PLC* Posterior lateral corner, *QoL* Quality of life, *SD* Standard deviation, *Sport/Rec* Function in Sport and Recreation

**Table 5:** Post hoc comparisons: Postoperative KOOS between groups at 5-year follow-up

KOOS Sport/Rec 5 years				
	**ACLR + LM repair**	**ACLR + LM resection**	**ACLR + LM injury left in situ**	**ACLR + LM repair + LM resection**
**Isolated ACLR**	0.81	0.28	0.88	0.26
**ACLR + LM repair**	-	0.51	0.91	0.21
**ACLR + LM resection**	-	-	0.50	0.29
**ACLR + LM injury left in situ**	-	-	-	0.28
**KOOS Symptoms 5 years**				
** Isolated ACLR**	0.17	0.11	0.64	0.18
**ACLR + LM repair**	-	0.49	0.41	0.13
**ACLR + LM resection**	-	-	0.71	0.14
**ACLR + LM injury left in situ**	-	-	-	0.18
**KOOS Pain 5 years**				
**Isolated ACLR**	0.90	0.81	0.69	0.25
**ACLR + LM repair**	-	0.86	0.73	0.28
**ACLR + LM resection**	-	-	0.78	0.29
**ACLR + LM injury left in situ**	-	-	-	0.28
**KOOS ADL 5 years**				
**Isolated ACLR**	0.51	0.46	0.74	0.33
**ACLR + LM repair**	-	0.71	0.72	0.41
**ACLR + LM resection**	-	-	0.98	0.38
**ACLR + LM injury left in situ**	-	-	-	0.36
**KOOS QoL 5 years**				
**Isolated ACLR**	0.41	0.73	0.64	0.74
**ACLR + LM repair**	-	0.50	0.69	0.50
**ACLR + LM resection**	-	-	0.82	0.73
**ACLR + LM injury left in situ**	-	-	-	0.80

*ACLR* Anterior cruciate ligament reconstruction, *ADL* Activities of daily living, *CI* Confidence Interval, *KOOS* Knee injury and Osteoarthritis Outcome Score, *LM* lateral meniscus, *QoL* Quality of life, *SD* Standard deviation, *Sport/Rec* Function in Sport and Recreation

### Postoperative KOOS by Lateral Meniscal Status 10 years after ACLR

Table 6 shows KOOS results at 10-year follow-up across LM treatment methods. The overall 10-year follow-up data was available for 11,092 patients (34.9%). No significant differences were found between isolated ACLR or the four LM treatment methods for four of five (4/5) KOOS subscales: Sports/Rec, Pain, ADL, and QoL. Significant difference was seen for the KOOS Symptoms subscale (*p* = 0.0095). When post hoc comparisons were performed across LM status for the KOOS Symptoms subscale, ACLR + LM resection (80.4 ± 18.0) had significantly lower scores than isolated ACLR (82.3 ± 17.6, *p* = 0.041). All other post hoc comparisons were statistically insignificant (Table 7).

**Table 6:** Post-operative KOOS at 10-year follow-up by lateral meniscal status

	**Isolated ACLR** **(*n* = 8,803)**	**ACLR + LM repair** **(*n* = 145)**	**ACLR + LM resection** **(*n* = 1,636)**	**ACLR + LM injury left in situ** **(*n* = 500)**	**ACLR + LM repair + LM resection** **(*n* = 8)**	***P***
**Sport/Rec**	69.3 ± 28.1 75 (0–100) *n* = 3,510	63.4 ± 31.5 60 (0–100) *n* = 51	68.2 ± 27.1 75 (0–100) *n* = 599	70.1 ± 27.2 80 (0–100) *n* = 184	82.0 ± 20.8 85 (50–100) *n* = 5	0.35
**Symptoms**	82.3 ± 17.6 85.7 (0–100) *n* = 3,516	79.1 ± 14.3 78.6 (46.4–100) *n* = 51	80.4 ± 18.0 85.7 (7.1–100) *n* = 600	81.6 ± 17.4 85.7 (21.4–100) *n* = 184	90.7 ± 5.4 89.3 (85.7–96.4) *n* = 5	**0.0095**
**Pain**	87.5 ± 15.7 94.4 (0–100) *n* = 3,515	86.1 ± 13.9 88.9 (41.7–100) *n* = 51	87.0 ± 14.9 91.7 (19.4–100) *n* = 599	87.7 ± 14.5 93.1 (30.6–100) *n* = 184	97.8 ± 3.6 100 (91.7–100) *n* = 5	0.088
**ADL**	92.5 ± 13.2 98.5 (0–100) *n* = 3,510	92.7 ± 11.0 97.1 (50–100) *n* = 51	92.6 ± 12.4 98.5 (25–100) *n* = 599	93.0 ± 12.8 98.5 (27.9–100) *n* = 184	99.1 ± 1.3 100 (97.1–100) *n* = 5	0.54
**QoL**	68.0 ± 24.8 75 (0–100) *n* = 3,508	61.0 ± 24.7 68.8 (0–100) *n* = 51	66.2 ± 24.5 68.8 (0–100) *n* = 598	68.7 ± 23.9 75 (12.5–100) *n* = 184	68.8 ± 21.2 81.3 (37.5–87.5) *n* = 5	0.060

Values are mean ± SD and median (minimum–maximum)

The total amount of follow-up data may vary within the KOOS subgroups. Thus, the numbers in the headline refer to the total count of patients corresponding follow-up data. Within the various KOOS subgroups, the numbers encompass the count of patients who completed their respective KOOS subgroup questionnaires

*ACL* Anterior cruciate ligament, *ACLR* Anterior cruciate ligament reconstruction, *ADL* Activities of daily living, *KOOS* Knee injury and Osteoarthritis Outcome Score, *LCL* Lateral collateral ligament, *LM* Lateral meniscus, *MCL* Medial collateral ligament, *PLC* Posterior lateral corner, *QoL* Quality of life, *SD* Standard deviation, *Sport/Rec* Function in Sport and Recreation

**Table 7:** Post hoc comparisons: Postoperative KOOS between groups at 10-year follow-up

KOOS Sport/Rec 10 years				
	**ACLR + LM repair**	**ACLR + LM resection**	**ACLR + LM injury left in situ**	**ACLR + LM repair + LM resection**
**Isolated ACLR**	0.24	0.72	0.63	0.29
**ACLR + LM repair**	-	0.27	0.22	0.21
**ACLR + LM resection**	-	-	0.51	0.25
**ACLR + LM injury left in situ**	-	-	-	0.38
**KOOS Symptoms 10 years**^**a**^				
**Isolated ACLR**	0.28	**0.041**	0.65	0.25
**ACLR + LM repair**	-	0.65	0.55	0.068
**ACLR + LM resection**	-	-	0.52	0.22
**ACLR + LM v**	-	-	-	0.25
**KOOS Pain 10 years**^**b**^				
**Isolated ACLR**	0.68	0.72	0.78	**0.040**
**ACLR + LM repair**	-	0.73	0.65	**0.039**
**ACLR + LM resection**	-	-	0.63	**0.018**
**ACLR + LM injury left in situ**	-	-	-	0.054
**KOOS ADL 10 years**				
**Isolated ACLR**	0.71	0.46	0.56	0.12
**ACLR + LM repair**	-	0.87	0.85	0.11
**ACLR + LM resection**	-	-	0.90	0.11
**ACLR + LM injury left in situ**	-	-		0.20
**KOOS QoL 10 years**				
**Isolated ACLR**	0.070	0.17	0.66	1.00
**ACLR + LM repair**	-	0.17	0.064	0.55
**ACLR + LM resection**	-	-	0.26	0.88
**ACLR + LM injury left in situ**	-	-	-	1.00

*ACLR* Anterior cruciate ligament reconstruction, *ADL* Activities of daily living, *CI* Confidence Interval, *KOOS* Knee injury and Osteoarthritis Outcome Score, *LM* Lateral meniscus, *QoL* Quality of life, *SD* Standard deviation, *Sport/Rec* Function in Sport and Recreation

^a^Difference between groups mean (95% CI) and effect size were calculated for the KOOS Symptoms. For Isolated ACLR and ACLR + LM resection: 95% CI, 1.93 (0.37; 3.52); Effect size, 0.109

^b^Difference between groups mean (95% CI) and effect size were calculated for the KOOS Pain. For Isolated ACLR and ACLR + LM repair + LM resection: 95% CI -10.3 (-12.7; -7.0); Effect size, 0.655. For ACLR + LM repair and ACLR + LM repair + LM resection: 95% CI, -11.7 (-16.5; -6.9); Effect size, 0.876. For ACLR + LM resection and ACLR LM repair + LM resection: 95% CI, -10.8 (-13.6; -7.4); Effect size, 0.730

## Discussion

The main findings of this study were that type of LM intervention in the setting of primary ACLR statistically affected postoperative KOOS scores. However, the difference found in the KOOS subscales falls below minimal clinical important difference (MCID) [29].

At 2-year follow-up, KOOS values from this study are comparable with the literature, particularly for isolated ACLR, ACLR + LM repair, and ACLR + LM resection [19, 20, 30–32]. This supports the current study’s results and adds further information to the body of literature on functional knee recovery after ACLR. No significant differences were found between isolated ACLR or the four LM treatment methods in 4 of 5 subscales of the KOOS (Sports/Rec, Pain, ADL, QoL). ACLR + LM repair had significantly lower mean KOOS Symptoms scores than isolated ACLR and ACLR + LM injury left in situ for this follow-up timepoint. While some subjective knee scores may be slower to recover after ACLR with meniscal repair compared to isolated ACLR and ACLR with meniscal resection [18], numerous studies have shown the long term benefit of repairing meniscal lesions in the setting of ACLR, resulting in improved QoL and decreased risk of osteoarthritis development [30, 33–35]. Yet, in this study, mean KOOS Symptoms scores were significantly lower for the ACLR + LM repair group compared to isolated ACLR and ACLR + LM injury left in situ groups. It is possible that the nature of non-surgically treated LM tears in this study was different compared to surgically treated LM injuries. Tears left in situ in this study may have been partial or non-traumatic [17, 36], having less impact on postoperative course and thus managed without intervention. However, a 2016 study calculating patient acceptable symptom state (PASS) values for KOOS subscales of patients 1 to 5 years postoperatively after ACLR found PASS for KOOS Symptoms subscale to be 57.1 (sensitivity 78%, specificity 67%) [27, 37]. At 2-year follow up in this current study, mean KOOS Symptoms subscale scores in the isolated ACLR as well as ACLR with all concomitant LM treatment methods were greater than the PASS value of 57.1. Furthermore, a MCID value for KOOS Symptoms subscale has been calculated at the threshold of 15.7 for early follow-up after ACLR [29]. When examining the comparisons with statistically significance in this study, differences in mean values between isolated ACLR and ACLR + LM repair as well as ACLR + LM injury left in situ were 2.3, much lower than previously reported MCID for the subscale score. However, wide standard deviations exist, suggesting that larger differences may have been present between some patients in the various treatment groups. Thus, the question remains on the clinical meaningfulness of the statistical findings of KOOS Symptoms subscale scores in the current study, as it is uncertain whether the small statistical differences between isolated ACLR, ACLR + LM repair, and ACLR + LM injury left in situ correlate with any meaningful clinical difference [38, 39]. It is possible that statistical significance has been achieved primarily due to large sample size, while the absolute difference between the groups may not be clinically meaningful to the patient.

At 5-year follow-up, significant differences were found between isolated ACLR and the four LM treatment methods in in KOOS Symptoms subscale. However, post hoc comparisons (comparing CI) showed no statistically significant differences between the groups. Additionally, although no direct comparisons were made between follow-up time points, KOOS subscale outcomes appeared to be preserved between 2- and 5-year follow up for isolated ACLR and ACLR with all LM treatment methods, suggesting the sustainability of each treatment option in terms of KOOS outcomes overtime.

At 10-year follow-up, no significant differences were found between isolated ACLR or the four LM treatment methods in 4 of 5 subscales of the KOOS. However, post hoc comparisons revealed that mean KOOS Symptoms for ACLR + LM resection had significantly lower scores than isolated ACLR. The risk of osteoarthritis after both ACLR and partial meniscal resection is well established [14, 40, 41], and a recent meta-analysis reported that although the risk of osteoarthritis development after ACLR exists, concomitant partial meniscal resection increases that risk of osteoarthritis development [42]. It is possible that patients undergoing ACLR + LM resection developed more symptomatic arthritis than those with isolated ACLR, thus accounting for the significant difference in KOOS symptom subscale scores at 10-year follow-up. These differences may reflect the clinical importance of protecting the LM in the setting of ACLR, as the value of meniscal preservation on long term knee health is widely agreed upon [43–46]. However, with an absolute difference of less than 2 points between the Symptoms subscale scores between the two groups, it again is uncertain that this difference is associated with any clinically significance [38, 39, 47] Thus, more research is needed to determine PASS values as well as MCID values at 10-year follow-up in order to aid in better understanding differences in KOOS subscale scores overtime in patients undergoing ACLR.

The results of this study provide valuable information on postoperative KOOS after isolated ACLR and ACLR with various treatments of LM lesions at short-, mid-, and long-term follow-up points in a large sample size of patients. Although this is just one data point, surgeons can utilize this information on functional knee recovery when considering options to address LM injury in the setting ACLR and when educating patients on expectations in the pre- and postoperative periods.

Lastly, the study has several limitations. Despite the large sample size and long-term follow-up, this cohort study from a registry of patients is a limitation as it includes patients undergoing operations at various centers with differing surgical techniques, rehabilitation guidelines, and quality of rehabilitation. Additionally, the follow-up data was limited, specifically at the 10-year follow-up timepoint leading to possible limitations when comparing the outcomes between the different treatment groups. In this study, only the KOOS was used to assess knee function after ACLR. Although widely used to assess knee function in patients after ACLR, the KOOS may not address all important functional limitation related to ACLR [48–50] and therefore, the KOOS may not fully capture all the important functional limitations related to ACLR. Also, no data was available on indications for choice of LM treatment, thus, it is unclear why some of the tears were decided to be repaired, resected, or treated nonoperatively. No data regarding the type or location of LM tear, and proportion of LM resected, were available either leading to some limitations as differing severities of LM injury as well as surgeon expertise, training, and clinical decision-making may affect the outcomes. Lastly, the presence of other injuries in addition to LM injuries, such as cartilaginous and other non-surgically treated ligament injuries, as well as possible revision surgeries during the follow-up period, may have influenced the postoperative outcomes.

## Conclusion

The results of this study suggest that LM injury during ACLR is associated with lower KOOS scores, particularly in the Symptoms subscale, at short- and long-term follow-up. However, this finding falls below MCID and therefore may not be clinically relevant [29].

### Supplementary Information

**Additional file 1: Table s1.** Baseline characteristics of the included patients with 2-year follow-up data. **Table s2.** Baseline characteristics of the included patients with 5-year follow-up data. **Table s3.** Baseline characteristics of the included patients with 10-year follow-up data.

## Data Availability

The dataset analyzed during the current study are available from the corresponding author on reasonable request.
