# Supplementary material for: Differences in postoperative knee function based on concomitant treatment of lateral meniscal injury in the setting of primary ACL reconstruction
Source: BMC Musculoskelet Disord. 2023 Sep 15;24:737. doi: 10.1186/s12891-023-06867-z (PMC10503181; doi:10.1186/s12891-023-06867-z)
Supplement: Supplementary file 1 — Additional file 1: Table s1. Baseline characteristics of the included patients with 2-year follow-up data. Table s2. Baseline characteristics of the included patients with 5-year follow-up data. Table s3. Baseline characteristics of the included patients with 10-year follow-up data. [file 12891_2023_6867_MOESM1_ESM.docx]

**Table s1. Baseline characteristics of the included patients with 2-year follow-up data.**

| Variable | Total  (n=29,378) | Isolated ACLR  (n=22,402) | ACLR + LM repair  (n=902) | ACLR + LM resection (n=4,763) | ACLR + LM injury left in situ  (n=1,249) | ACLR + LM repair + LM resection  (n=62) |
| --- | --- | --- | --- | --- | --- | --- |
| Age at the time of injury (years) | 25.5 ± 9.5  23 (1-70) | 25.9 ± 9.8  23 (1-70) | 22.3 ± 8.3  20 (1-57) | 24.6 ± 8.7  22 (7-64) | 24.1 ± 8.5  21 (11-58) | 21.0 ± 6.4  19 (11-39 |
| Age at the time of surgery (year) | 27.1 ± 9.8  24 (15-71) | 27.5 ± 10.0  25 (15-71) | 23.7 ± 8.6  21 (15-58) | 26.2 ± 9.0  24 (15-66) | 25.3 ± 8.9  23 (15-60) | 21.9 ± 6.4  19 (15-42) |
| Sex (male) | 16,562 (56.4) | 12,066 (53.9) | 516 (57.2) | 3,229 (67.8) | 706 (56.5) | 45 (72.6) |
| BMI (kg/m^2^) | 24.5 ± 3.3  24.2 (15.4-49.8) | 24.5 ± 3.3  24.1 (15.4-49.8) | 23.7 ± 2.8  23.4 (17.4-35.4) | 24.8 ± 3.2  24.4 (16-42.7) | 24.4 ± 3.3  23.9 (17.8-44.1) | 23.8 ± 2.9  23.3 (19.8-29.6) |
| Smoking (yes) | 759 (4.8) | 586 (4.9) | 26 (4.9) | 112 (4.3) | 33 (5.0) | 2 (5.4) |
| Time from injury to surgery (months) | 17.3 ± 31.0  7.6 (0-458.4) | 17.6 ± 31.5  7.8 (0-458.4) | 14.3 ± 27.8  6.1 (0.1-247.9) | 17.4 ± 30.5  7.4 (0.1-434.9) | 14.5 ± 26.7  7.2 (0.1-358.9) | 10.2 ± 20.0  5.3 (0.8-140.8) |
| ACL graft (yes) |  |  |  |  |  |  |
| Patellar tendon autograft | 1,659 (5.7) | 1,284 (5.8) | 50 (5.6) | 260 (5.5) | 61 (4.9) | 4 (6.5) |
| Semitendinosus autograft | 26,887 (92.6) | 20,506 (92.7) | 804 (90.2) | 4,367 (92.6) | 1,158 (93.5) | 52 (83.9) |
| Quadriceps tendon autograft | 378 (1.3) | 264 (1.2) | 25 (2.8) | 73 (1.5) | 11 (0.9) | 5 (8.1) |
| Allograft | 58 (0.2) | 37 (0.2) | 8 (0.9) | 10 (0.2) | 3 (0.2) | 0 (0.0) |
| Direct suture/synthetic/other | 50 (0.2) | 36 (0.2) | 4 (0.4) | 4 (0.1) | 5 (0.4) | 1 (1.5) |
| Concomitant injury except meniscal injury (yes) |  |  |  |  |  |  |
| Cartilaginous injury (yes) |  |  |  |  |  |  |
| Lateral femoral condyle | 1,454 (4.9) | 778 (3.5) | 70 (7.8) | 508 (10.7) | 86 (6.9) | 12 (19.4) |
| Medial femoral condyle | 3,943 (13.4) | 2,891 (12.9) | 144 (16.0) | 735 (15.4) | 156 (12.5) | 17 (27.4) |
| Lateral patella | 651 (2.2) | 460 (2.1) | 27 (3.0) | 135 (2.8) | 26 (2.1) | 3 (4.8) |
| Medial patella | 1,184 (4.0) | 869 (3.9) | 33 (3.7) | 210 (4.4) | 65 (5.2) | 7 (11.3) |
| Lateral tibial plateau | 1,638 (5.6) | 960 (4.3) | 82 (9.1) | 481 (10.1) | 193 (15.5) | 13 (21.0) |
| Medial tibial plateau | 1,007 (3.4) | 774 (3.5) | 24 (2.7) | 179 (3.8) | 27 (2.2) | 3 (4.8) |
| Trochlea | 693 (2.4) | 484 (2.2) | 35 (3.9) | 142 (3.0) | 29 (2.3) | 3 (4.8) |
| Collateral ligament injury (yes) |  |  |  |  |  |  |
| LCL | 234 (0.8) | 151 (0.7) | 14 (1.6) | 50 (1.0) | 18 (1.4) | 1 (1.6) |
| MCL | 1,087 (3.7) | 20,506 (92.79 | 804 (90.2) | 4,367 (92.6) | 1,158 (93.5) | 52 (83.9) |
| PLC injury (yes) | 23 (0.1) | 15 (0.1) | 2 (0.2) | 6 (0.1) | 0 (0.0) | 0 (0.0) |
| Activity at the time of injury (yes) |  |  |  |  |  |  |
| Alpine/skiing | 4,265 (14.5) | 3,475 (15.6) | 128 (14.2) | 489 (10.3) | 165 (13.2) | 8 (12.9) |
| Pivoting sport | 19,922 (68.0) | 14,852 (66.5) | 645 (71.6) | 3,482 (73.2) | 896 (71.8) | 47 (75.8) |
| Non-pivoting sport | 1,204 (4.1) | 887 (4.0) | 37 (4.1) | 217 (4.6) | 61 (4.9) | 2 (3.2) |
| Other physical activity | 1,082 (3.7) | 857 (3.89 | 22 (2.4) | 158 (3.3) | 43 (3.4) | 2 (3.2) |
| Traffic-related | 465 (1.6) | 344 (1.5) | 19 (2.1) | 84 (1.8) | 17 (1.4) | 1 (1.6) |
| Other | 2,379 (8.1) | 1,931 (8.6) | 50 (5.5) | 330 (6.9) | 66 (5.3) | 2 (3.2) |

Values are given as n (%) and mean ± SD or median (minimum-maximum) for categorical and continuous as well as ordinal variables, respectively. The sums may vary to because of missing values. The variables with missing values, n (%) of the total sample were Age at the time of injury 598 (2.0), BMI 13,797 (47.0), Smoking 12,558 (46.2), Time from injury to surgery 634 (2.2), Cartilaginous injury 22,968 (78.2), ACL graft 346 (1.2), and Activity at the time of injury 61 (0.2). ACL=anterior cruciate ligament; ACLR=anterior cruciate ligament reconstruction; BMI=body mass index; LCL=lateral collateral ligament; LM=lateral meniscus; MCL=medial collateral ligament; PLC=posterior lateral corner; SD=standard deviation.

Pivoting sport (American football/rugby, basketball, dancing, floorball, gymnastics, handball, ice hockey/bandy, martial arts, racket sports, soccer, volleyball, wrestling); Non-pivoting sport (cross-country skiing, cycling, horseback riding, motocross/endure, skateboarding, snowboarding, and surfing/wakeboarding); Alpine/skiing; Other physical activity (other recreational sport, exercise, trampoline); Traffic related, and Other (other outdoor activity and work).

**Table s2. Baseline characteristics of the included patients with 5-year follow-up data.**

| Variable | Total  (n=22,291) | Isolated ACLR  (n=17,195) | ACLR + LM repair  (n=509) | ACLR + LM resection (n=3,603) | ACLR + LM injury left in situ  (n=954) | ACLR + LM repair + LM resection  (n=30) |
| --- | --- | --- | --- | --- | --- | --- |
| Age at the time of injury (years) | 25.3 ± 9.3  23 (5-70) | 25.6 ± 9.5  23 (5-70) | 21.9 ± 8.3  19 (7-57) | 24.4 ± 8.5  22 (9-62) | 23.8 ± 8.4  21 (11-58) | 20.5 ± 5.9  19 (11-38) |
| Age at the time of surgery (year) | 26.9 ± 9.6  24 (15-71) | 27.2 ± 9.8  25 (15-71) | 23.2 ± 8.7  21 (15-58) | 26.0 ± 8.9  24 (15-63) | 25.0 ± 8.7  22 (15-60) | 21.7 ± 6.1  19.5 /15-42) |
| Sex (male) | 12,757 (57.2) | 9,442 (54.9) | 293 (57.6) | 2,462 (68.3) | 536 (56.2) | 24 (80.0) |
| BMI (kg/m^2^) | 24.6 ± 3.3  24.2 (16-49.8) | 24.6 ± 3.3  24.2 (16-49.8) | 23.8 ± 2.9  23.3 (18.2-35.4) | 24.9 ± 3.2  24.4 (16-42.7) | 24.4 ± 3.3  23.8 (17.8-44.1) | 24.0 ± 2.8  23.4 (20.8-29.6) |
| Smoking (yes) | 612 (5.1) | 481 (5.2) | 21 (6.7) | 82 (4.2) | 27 (5.3) | 1 (5.6) |
| Time from injury to surgery (months) | 17.8 ± 31.1  8 (0-452.4) | 18.1 ± 31.5  8.2 (0-452.4) | 13.5 ± 25.7  6.1 (0.1-245.2) | 17.8 ± 31.2  7.7 (0.1-434.9) | 14.3 ± 26.0  7.4 (0.1-358.9) | 13.6 ± 27.9  5.2 (0.8-140.8) |
| ACL graft (yes) |  |  |  |  |  |  |
| Patellar tendon autograft | 1,286 (5.8) | 1,013 (6.0) | 29 (5.8) | 193 (5.4) | 49 (5.2) | 2 (6.7) |
| Semitendinosus autograft | 20,537 (93.2) | 15,830 (93.2) | 455 (90.8) | 3,338 (93.5) | 888 (94.0) | 26 (86.7) |
| Quadriceps tendon autograft | 120 (0.5) | 81 (0.5) | 9 (1.8) | 26 (0.7) | 3 (0.3) | 1 (3.3) |
| Allograft | 45 (0.2) | 29 (0.2) | 5 (1.0) | 9 (0.3) | 2 (0.2) | 0 (0.0) |
| Direct suture/synthetic/other | 38 (0.2) | 28 (0.2) | 3 (0.6) | 3 (0.1) | 3 (0.3) | 1 (3.3) |
| Concomitant injury except meniscal injury (yes) |  |  |  |  |  |  |
| Cartilaginous injury (yes) |  |  |  |  |  |  |
| Lateral femoral condyle | 1,120 (5.0) | 612 (3.6) | 37 (7.3) | 396 (10.2) | 69 (7.2) | 6 (20) |
| Medial femoral condyle | 3,032 (13.6) | 2,268 (13.2) | 78 (15.3) | 555 (15.4) | 119 (12.5) | 12 (40) |
| Lateral patella | 502 (2.3) | 356 (2.1) | 15 (2.9) | 109 (3.0) | 21 (2.2) | 1 (3.3) |
| Medial patella | 916 (4.1) | 672 (3.9) | 18 (3.5) | 169 (4.7) | 52 (5.4) | 5 (16.7) |
| Lateral tibial plateau | 1,219 (5.5) | 743 (4.3) | 50 (9.8) | 352 (9.8) | 66 (6.9) | 8 (26.7) |
| Medial tibial plateau | 830 (3.7) | 644 (3.7) | 14 (2.8) | 147 (4.1) | 23 (2.4) | 2 (6.7) |
| Trochlea | 518 (2.3) | 369 (2.1) | 16 (3.1) | 106 (2.9) | 25 (2.6) | 2 (6.7) |
| Collateral ligament injury (yes) |  |  |  |  |  |  |
| LCL | 164 (0.7) | 107 (0.6) | 7 (1.4) | 40 (1.1) | 9 (0.9) | 1 (3.3) |
| MCL | 747 (3.4) | 511 (3.0) | 44 (8.6) | 136 (3.8) | 55 (5.8) | 1 (3.3) |
| PLC injury (yes) | 21 (0.1) | 13 (0.1) | 2 (0.4) | 6 (0.2) | 0 (0.0) | 0 (0.0) |
| Activity at the time of injury (yes) |  |  |  |  |  |  |
| Alpine/skiing | 3,007 (13.5) | 2,467 (14.4) | 70 (13.8) | 349 (9.7) | 116 (12.2) | 5 (16.7) |
| Pivoting sport | 15,254 (68.6) | 11,540 (67.3) | 362 (71.3) | 2,639 (73.3) | 692 (72.6) | 21 (70.0) |
| Non-pivoting sport | 930 (4.2) | 687 (4.0) | 19 (3.7) | 172 (4.8) | 51 (5.4) | 1 (3.3) |
| Other physical activity | 826 (3.7) | 666 (3.9) | 12 (2.4) | 117 (3.3) | 30 (3.1) | 1 (3.3) |
| Traffic-related | 382 (1.7) | 289 (1.7) | 12 (2.4) | 66 (1.8) | 15 (1.6) | 0 (0.0) |
| Other | 1,831 (82.) | 1,490 (8.7) | 33 (6.5) | 257 (7.1) | 49 (5.1) | 2 (6.7) |

Values are given as n (%) and mean ± SD or median (minimum-maximum) for categorical and continuous as well as ordinal variables, respectively. The sums may vary to because of missing values. The variables with missing values, n (%) of the total sample were Age at the time of injury 525 (2.4), BMI 10,460 (46.9), Smoking 10,304 (46.2), Time from injury to surgery 555 (2.5), Cartilaginous injury 17,396 (78.0), ACL graft 265 (1.2), and Activity at the time of injury 61 (0.3). ACL=anterior cruciate ligament; ACLR=anterior cruciate ligament reconstruction; BMI=body mass index; LCL=lateral collateral ligament; LM=lateral meniscus; MCL=medial collateral ligament; PLC=posterior lateral corner; SD=standard deviation.

Pivoting sport (American football/rugby, basketball, dancing, floorball, gymnastics, handball, ice hockey/bandy, martial arts, racket sports, soccer, volleyball, wrestling); Non-pivoting sport (cross-country skiing, cycling, horseback riding, motocross/endure, skateboarding, snowboarding, and surfing/wakeboarding); Alpine/skiing; Other physical activity (other recreational sport, exercise, trampoline); Traffic related, and Other (other outdoor activity and work).

**Table s3. Baseline characteristics of the included patients with 10-year follow-up data.**

| Variable | Total  (n=11,092) | Isolated ACLR  (n=8,803) | ACLR + LM repair  (n=145) | ACLR + LM resection (n=1,636) | ACLR + LM injury left in situ  (n=500) | ACLR + LM repair + LM resection  (n=8) |
| --- | --- | --- | --- | --- | --- | --- |
| Age at the time of injury (years) | 25.1 ± 9.1  23 (5-64) | 25.4 ± 9.3  23 (5-64) | 22.0 ± 9.2  18.5 (7-57) | 24.2 ± 8.4  22 (11-61) | 23.7 ± 8.3  21 (11-54) | 20.5 ± 4.9  20 (15-28) |
| Age at the time of surgery (year) | 26.9 ± 9.5  25 (15-65) | 27.2 ± 9.6  25 (15-65) | 23.9 ± 10.0  20 (15-58) | 26.0 ± 8.9  24 (15-62) | 24.8 ± 8.5  22 (15-55) | 21.2 ± 5.0  21 (15-29) |
| Sex (male) | 6,390 (57.6) | 4,911 (55.8) | 77 (53.1) | 1,109 (67.8) | 287 (57.4) | 6 (75.0) |
| BMI (kg/m^2^) | 24.7 ± 94.8  24.2 (16.7-44.8) | 24.7 ± 3.3  24.2 (16.7-44.8) | 23.5 ± 2.4  22.9 (19.4-30.4) | 24.9 ± 3.2  24.4 (17.2-39.2) | 24.1 ± 2.9  23.7 (17.8-34) | 22.4 ± 2.8  21.1 (20.8-26.5) |
| Smoking (yes) | 272 (5.2) | 219 (5.4) | 3 (3.9) | 35 (4.5) | 15 (6.0) | 0 (0.0) |
| Time from injury to surgery (months) | 19.1 ± 31.3  8.9 (0-405.6) | 19.5 ± 31.9  9 (0-405.6) | 16.0 ± 28.4  6.1 (0.1-183.3) | 19.0 ± 29.6  9.1 (0.1-349.2) | 13.8 ± 25.9  7.7 (0.3-358.9) | 7.28 ± 4.14  6.24 (3.02-16.2) |
| ACL graft (yes) |  |  |  |  |  |  |
| Patellar tendon autograft | 991 (9.0) | 791 (9.1) | 11 (7.7) | 143 (8.8) | 22 (8.9) | 2 (25.0) |
| Semitendinosus autograft | 9,947 (90.6) | 7,896 (90.6) | 128 (89.5) | 1,469 (90.8) | 448 (90.3) | 6 (75.0) |
| Quadriceps tendon autograft | 12 (0.1) | 7 (0.1) | 1 (0.7) | 3 (0.2) | 1 (0.2) | 0 (0.0) |
| Allograft | 18 (0.2) | 14 (0.2) | 14 (0.2) | 1 (0.1) | 2 (0.4) | 0 (0.0) |
| Direct suture/synthetic/other | 16 (0.1) | 12 (0.1) | 12 (0.1) | 1 (0.1) | 1 (0.2) | 0 (0.0) |
| Concomitant injury except meniscal injury (yes) |  |  |  |  |  |  |
| Cartilaginous injury (yes) |  |  |  |  |  |  |
| Lateral femoral condyle | 561 (5.1) | 330 (3.7) | 13 (9.0) | 180 (11.0) | 36 (7.2) | 2 (0.25) |
| Medial femoral condyle | 1,639 (14.8) | 1,274 (14.5) | 28 (19.3) | 268 (16.4) | 65 (13.0) | 4 (50.0) |
| Lateral patella | 298 (2.7) | 216 (2.5) | 9 (6.2) | 61 (3.7) | 12 (2.4) | 0 (0.0) |
| Medial patella | 526 (4.7) | 402 (4.6) | 11 (7.6) | 86 (5.3) | 27 (5.4) | 0 (0.0) |
| Lateral tibial plateau | 630 (5.7) | 406 (4.6) | 15 (10.3) | 173 (10.6) | 35 (7.0) | 1 (12.5) |
| Medial tibial plateau | 482 (4.3) | 390 (4.4) | 9 (6.2) | 71 (4.3) | 11 (2.2) | 1 (12.5) |
| Trochlea | 277 (2.5) | 202 (2.3) | 9 (6.2) | 51 (3.1) | 15 (3.0) | 0 (0.0) |
| Collateral ligament injury (yes) |  |  |  |  |  |  |
| LCL | 90 (0.8) | 58 (0.7) | 2 (1.4) | 24 (1.5) | 5 (1.0) | 1 (12.5) |
| MCL | 369 (3.3) | 270 (3.1) | 12 (8.3) | 51 (3.1) | 36 (7.2) | 0 (0.0) |
| PLC injury (yes) | 13 (0.1) | 10 (0.1) | 0 (0.0) | 3 (0.2) | 0 (0.0) | 0 (0.0) |
| Activity at the time of injury (yes) |  |  |  |  |  |  |
| Alpine/skiing | 1,417 (12.8) | 1,170 (13.4) | 23 (16.0) | 160 (9.8) | 62 (12.4) | 2 (25.0) |
| Pivoting sport | 7,582 (68.7) | 5,938 (67.9) | 94 (65.3) | 1,185 (72.6) | 359 (71.9) | 6 (75.0) |
| Non-pivoting sport | 461 (4.2) | 350 (4.0) | 6 (4.2) | 75 (4.6) | 30 (6.0) | 0 (0.0) |
| Other physical activity | 454 (4.1) | 375 (4.3) | 3 (2.1) | 59 (3.6) | 17 (3.4) | 0 (0.0) |
| Traffic-related | 204 (1.8) | 153 (1.7) | 6 (4.2) | 35 (2.1) | 10 (2.0) | 0 (0.0) |
| Other | 913 (8.3) | 761 (8.7) | 12 (8.3) | 119 (7.3) | 21 (4.2) | 0 (0.0) |

Values are given as n (%) and mean ± SD or median (minimum-maximum) for categorical and continuous as well as ordinal variables, respectively. The sums may vary to because of missing values. The variables with missing values, n (%) of the total sample were Age at the time of injury 385 (3.5), BMI 5,976 (53.9), Smoking 5,911(53.3), Time from injury to surgery 400 (3.6), Cartilaginous injury 8,464 (76.3), ACL graft 108 (1.0), and Activity at the time of injury 61 (0.5). ACL=anterior cruciate ligament; ACLR=anterior cruciate ligament reconstruction; BMI=body mass index; LCL=lateral collateral ligament; LM=lateral meniscus; MCL=medial collateral ligament; PLC=posterior lateral corner; SD=standard deviation.

Pivoting sport (American football/rugby, basketball, dancing, floorball, gymnastics, handball, ice hockey/bandy, martial arts, racket sports, soccer, volleyball, wrestling); Non-pivoting sport (cross-country skiing, cycling, horseback riding, motocross/endure, skateboarding, snowboarding, and surfing/wakeboarding); Alpine/skiing; Other physical activity (other recreational sport, exercise, trampoline); Traffic related, and Other (other outdoor activity and work).
